# Seismological evidence for a non-monotonic velocity gradient in the topmost outer core

**DOI:** 10.1038/srep08613

**Published:** 2015-02-27

**Authors:** Vivian Tang, Li Zhao, Shu-Huei Hung

**Affiliations:** 1 Institute of Earth Sciences, Academia Sinica, Taipei, Taiwan; 2 Department of Geosciences, National Taiwan University, Taipei, Taiwan

## Abstract

The Earth's core is mostly an Fe-Ni alloy with a fraction of light elements (~10 wt%, mainly O, S and Si). Accumulation of these light elements under the core-mantle boundary (CMB) may lead to chemical stratification. Seismic observations have been presented both for and against the stratification in the topmost region of the outer core. Here we investigate the structure under the CMB using differential travel times between SKKS and S3KS waves. We obtain 606 high-quality S3KS-SKKS differential travel times with global path coverage. Result from a Bayesian inversion of these differential times indicates that the seismic velocity in the top 800 km of the outer core is ~0.07% on average lower than that in model PREM. The depth-dependent velocity profile, in particular a low-velocity zone of up to ~0.25% lower than PREM at ~80 km below the CMB, strongly favors the existence of stratification at the top of the outer core.

The Earth's solid inner core consists mainly of heavy minerals Fe (~85%) and Ni (~6%) with a significant fraction of light elements such as O, S and Si^1^,^2^,^3^,^4^,^5^,^6^. The outer core, on the other hand, is liquid with similar composition as the inner core and has been considered to be more or less chemically homogenized by vigorous internal convection^7^. Variation in the structure of the outer core is thought of purely due to the depth gradient of pressure and temperature. This consideration is also the basis in establishing the outer core structure in the global average one-dimensional (1D) models such as PREM^8^. However, there have also been geodynamical studies suggesting that light materials may be trapped beneath the CMB, leading to an incomplete mixture of light and heavy materials and a chemical stratification at the top of the outer core^9^,^10^,^11^,^12^.

SKS and its core multiples S*m*KS provide us the best means to study the structure of the outer core. Although density alters the wave speed, the effect of lighter materials on the wave speed in the outer core is not straightforward. The elastic property of a multi-component liquid behaves non-linearly if the mixture has not reached an ideal state, and may offset the effect of density change on the wave speed. Therefore, chemical stratification at the top of the outer core due to the existence of lighter materials there could lead to the non-intuitive outcome of wave speed decrease^13^.

Deviations of wave speed from PREM near the top of the outer core have been reported and updated by various of groups using records with different geographical coverage. A majority of those studies report a sharper wave speed drop in a narrow zone (<200 km) towards the CMB than the smooth profile in PREM, suggesting an accumulation of light materials right beneath the CMB^14^,^15^,^16^,^17^,^18^,^19^. The latest seismic evidence supporting those observations has been from travel time inversions of S*m*KS waves along a few isolated paths under the Pacific Ocean, where the wave speed at the top of the core is 0.45% lower than PREM, and the anomaly gradually reduces to zero at 300 km below the CMB^20^,^21^. However, another inversion of globally sampled S*m*KS travel times found that PREM remains the best model in the 200-km layer immediately beneath the CMB^22^. The detailed structure at the top of the outer core is yet to be resolved.

## Measuring S3KS-SKKS differential travel times

**Figure 1:**
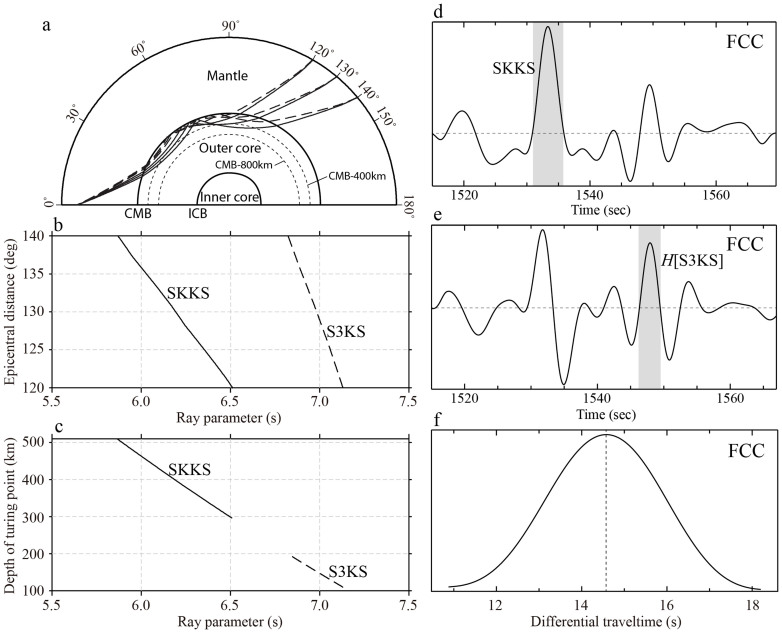
Ray theory properties (in PREM) and waveform processing of SKKS and S3KS waves. Ray paths are shown in (a) for SKKS (solid) and S3KS (dashed) at 120°, 130° and 140°, while (b) and (c) display respectively the epicentral distance and turning-point depth below CMB as functions of ray parameter for a source at 500-km depth. (d) Radial-component velocity record (0.005–0.2 Hz) at station FCC from the 25 July 2004 Sumatra earthquake (depth 600 km). (e) Hilbert transform of the waveform in (d). (f) Cross-correlation of the shaded segments of the waveforms in (d) and (e), the lag time at the maximum amplitude yields the S3KS-SKKS differential travel time.

In this study, we focus on the differential traveltimes between SKKS and S3KS waves in the distance range 120°–140° and from deep earthquakes (400 km and deeper) to avoid the interference of near-source surface reflections. Beyond 140° the S3KS is weaker and its waveform becomes more complex because of interfering S4KS and S5KS waves. The ray parameters of SKKS and S3KS waves in PREM vary in the ranges of 5.8 ~ 6.5 s/deg and 6.8 ~ 7.1 s/deg, respectively, in the distance range 120°–140° (Fig. 1b), and monotonically decrease with distance. The S3KS waves all turn in the top 200 km of the outer core, whereas the SKKS waves can reach down to ~500 km below the CMB (Fig. 1c).

Due to the rather long traveling distance, higher frequency content of the SKKS and S3KS waves is strongly attenuated. As a result, the SKKS and S3KS phases have relatively low signal-to-noise ratio (SNR) at short period, and their arrival times are difficult to define precisely. In order to ensure data quality, we measure the SKKS and S3KS differential traveltimes by cross-correlations of their recorded waveforms bandpass filtered between 0.005 and 0.2 Hz. Before cross-correlation a Hilbert transform is applied to the S3KS waveform to account for the π/2 phase shift from the extra caustic along the S3KS ray path in comparison to SKKS (Fig. 1e). The first Fresnel zone widths of SKKS and S3KS waves at the CMB are similar to that of SKS, which is well over 500 km at the CMB^23^, larger than the separation between the ray paths of SKKS and S3KS. As a result, the sensitivity of their differential travel time has a complicated pattern in the mantle (see Fig. S1 in Supplementary Information). Therefore, the mean property in a dataset of SKKS and S3KS differential times with globally distributed path coverage will be less prone to the influence of structural perturbations outside the core.

**Figure 2:**
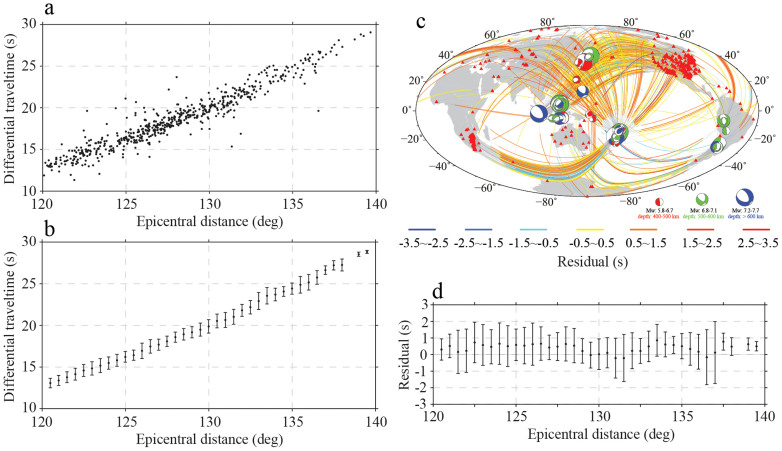
S3KS-SKKS differential travel time measurements. Raw measurements are plotted in (a), whereas (b) shows the measurements with outliers removed and averaged over 1°-bins at every 0.5° interval, and the vertical bars indicate the standard deviation. The residuals of the differential travel times relative to PREM are plotted along the corresponding source-receiver great-circle paths in (c), which was generated by the Generic Mapping Tools^36^. The residuals are plotted such that segments of the paths outside the core are in gray while those inside the core are in color. The color of a path indicates the residual value. The residuals of the binned observations in (b) relative to PREM predictions are plotted in (d).

We processed the waveforms of 78 earthquakes at 374 stations (Fig. 2c) and measured the SKKS and S3KS differential travel times. A total of 1519 measurements were obtained, from which 606 were retained for further analysis (Fig. 2a) after a rigorous examination of the waveforms (see Fig. S2 in Supplementary Information). We also obtained the corresponding differential travel times in model PREM by the same procedure using synthetic waveforms calculated by the direct-solution method^24^,^25^,^26^ (DSM). Comparison between observed and model-predicted differential travel times clearly suggests that the velocity at the top of the core in PREM needs to be lowered (Fig. 2d). In addition, significant scattering can also be seen in the differential travel times, which may be due to lateral heterogeneities in the lowermost mantle and/or the outer core^27^,^28^.

## Bayesian inversion of S3KS-SKKS differential travel times

We conducted an inversion of the observed SKKS and S3KS differential travel times (the raw data in Fig. 2a) for the vertical velocity profile below the CMB based on the Bayesian inference^29^ (see Methods). The model is parameterized as the wave speed perturbations from PREM at 40 evenly spaced radial samples in the top 800 km region of the outer core. We adopt a cosine-square tapered boxcar function for the *a priori* probability distribution of the model perturbation: a unity probability between −0.5% and +0.25% from PREM and zero otherwise, tapered by a cosine-squared function of depth so that the bounds of the model prior are largest at the CMB (between −0.5% and +0.25% from PREM) and diminishes smoothly to zero at 800 km below the CMB to avoid introducing any artificial discontinuities in the outer core. The 40-dimensional model space is sampled by a Markov Chain Monte Carlo (MCMC) sampler enhanced by the Metropolis-Hastings (MH) algorithm^30^,^31^,^32^. We use the standard deviations of the measurements in Fig. 2b for the data uncertainty in the Bayesian inversion.

Although the MCMC sampler enhanced by the MH algorithm provides an efficient scheme to make Bayesian inference of samples of the model space, the large number of model samples still prevent us from using the DSM to obtain model-predicted differential travel times. Here, we use the much more efficient TauP Toolkit^33^ to predict the differential traveltimes, and correct for the finite-frequency effect (see Methods).

**Figure 3:**
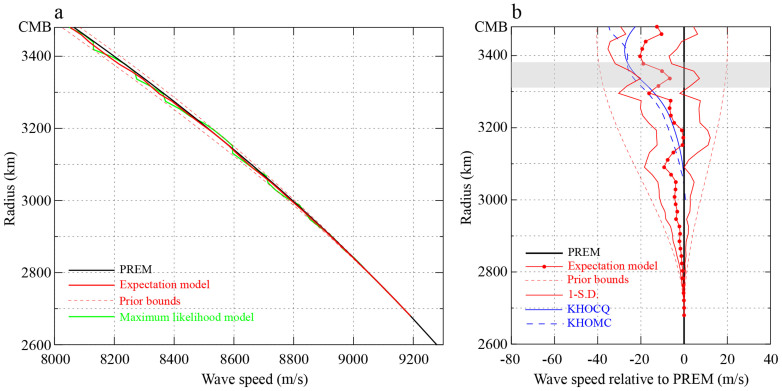
Topmost outer core structure from Bayesian inversion. (a) Wave speed in PREM (solid black line) and our expectation model (solid red line) from Bayesian inversion. Green line shows the model with the maximum likelihood, whereas the red dashed curves are the bounds of the model prior, i.e. the *a priori* probability distribution is unity inside the bounds and identically zero outside. (b) Perturbations of wave speed relative to PREM (solid black line). Shown here are our expectation model (dotted solid red line), one-standard deviation (red lines), and models KHOCQ^20^ (blue solid line) and KHOMC^21^ (blue dashed line) for comparison. Red dashed lines are bounds of the model prior.

One of the most commonly presented solutions from the Bayesian inversion is the expectation model, obtained by the ensemble averages of the model parameters weighted by the *a posteriori* probability distribution function. The expectation model and its standard deviation shown in Fig. 3 represents the ensemble average of 40,000 model samples. A series of bootstrap tests have also been conducted to validate the result (see Fig. S3 in Supplementary Information). There is clearly a strong depth-dependence in wave speed gradient. The wave speed is overall lower than PREM in the top 800 km of the outer core with an average velocity perturbation of −0.07% from PREM. The maximum departures from PREM is −0.25% at ~80 km below the CMB, whereas at ~300 km below the CMB the wave speed is closest to PREM. The narrow high-velocity zone (~70 km thick, shaded in Fig. 3b) sandwiched at ~150 km beneath the CMB is a very robust feature of the inversion. The wave speed approaches to that of PREM at ~550 km below the CMB where our data have lost structural sensitivity.

As shown in Fig. 3b, at long wavelength, our statistically-derived expectation model has a certain resemblance to the deterministic models KHOCQ^20^ and KHOMC^21^ which sample two paths beneath the Pacific Ocean. The relatively rapid variations of wave speed with depth at ~150 km (towards PREM) and ~400 km (away from PREM) below the CMB are very stable features in the Bayesian inversion, as is the structure at ~300 km below the CMB where the wave speed is closest to PREM.

## Geodynamical implications

The wave speed perturbation of up to −0.25% at ~80 km below the CMB violates the assumption of chemical homogeneity in the topmost outer core^34^, and implies the existence of compositional stratification. In the fluid outer core, the wave speed is determined by the density and bulk modulus. Intuitively, the introduction of light elements tends to increase the wave speed if the bulk modulus remains unchanged. However, the behavior of the bulk modulus in a liquid mixture of Fe-Ni with minor amount of O, S and Si depends on the level of mixing of the cocktail of elements. It has been demonstrated that the bulk modulus of the Fe-Ni alloy mixed non-ideally with light elements O, S and Si is very sensitive to the proportion of the light elements as well as the thermal-dynamical conditions^13^, and therefore the total effect on wave speed is rather complicated.

Light materials concentrating beneath the CMB may come from three different sources, with different geodynamical implications. Light elements are released from the inner core during its solidification, which has been proposed as the source of power that drives the geodynamo^35^. Nevertheless, it is unlikely that these light materials from the inner core have significant contribution to the compositional stratification near the top of the outer core for the simple reason that the bulk of the outer core is considered as chemically homogenized. Another proposal for the presence of light materials is that they already existed during the formation of the outer core in the early history of the Earth, and this layering of distinct composition has been maintained through barodiffusivity^12^. It has been shown that the wave speed profiles in the seismic models KHOCQ and KHOMC agree reasonably well with diffusion models having an initial chemically heterogeneous layer of ~200 km thick^34^. The third explanation for the presence of light materials below the CMB is that they are derived from the bottom of the mantle through mantle-core interaction^9^,^10^,^11^, which can sustain a chemically distinct layer of ~70 km in thickness at the top of the outer core.

Notwithstanding the support for the existence of a stable compositional layering below the CMB, the mechanisms provided by dynamical modelings do not fully reconcile with our velocity profile obtained from S3KS-SKKS differential traveltimes. The thickness of seismic structural heterogeneity is well over 400 km, and the deviation of wave speed from that of PREM is highly variable with depth. These two features defy the explanation of any single dynamical model. Irrespective of their origin, the excess light elements in the topmost part of the outer core lead to disruptions to the simple structural gradient due to pressure and temperature only. In fact, it can be surmised that the light elements at the top of the outer core come from multiple sources: both from an ancient existence when the core was formed and from the ongoing mantle-core interaction, resulting in wave speed perturbations that vary with depth.

In this study, our Bayesian inversion of the S3KS-SKKS differential travel times provides seismological constrains on the globally averaged wave speed profile in the topmost outer core. Our expectation model shows that the velocity gradient in the topmost part of the outer core has robust short-wavelength variations, strongly suggesting the existence of chemically-induced stratification. Although the alternating high and low velocity gradients may introduce more complexities in the interpretation of the seismic model, they also offer more detailed seismic constrains that challenge geodynamicists to come up with new models to satisfy them.

## Methods

We treat the inversion of S3KS-SKKS differential travel times for the vertical velocity structure in the top 800 km of the outer core as a non-linear inverse problem, which we solve by the Bayesian inference, i.e. we sample the model space and try to infer the model properties by inspecting the *a posteriori* probability distribution^29^
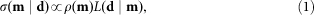
where *ρ*(**m**) is the *a priori* probability distribution of the model **m**, and *L*(**d** | **m**) is the likelihood function
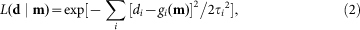
where **g**(**m**) is the model prediction, and *τ_i_* is the uncertainty of data *d_i_*. The likelihood indicates the probability of predicting data **d** by the model sample **m**.

The *a posteriori* probability distribution *σ*(**m** | **d**) represents the probability of the solution of the inverse problem being the model **m** under the constrains of the observed data **d** with standard deviation **τ** and the specified prior *ρ*(**m**). We choose the *a priori* probability distribution such that it is unity inside certain velocity range and zero outside. At each depth in our model, the velocity range is such that the perturbation is between −0.5% and +0.25% from the velocity in PREM. These bounding values are further tapered by a cosine-squared function of depth which has the value of one at the CMB and zero at 800 km below the CMB. The cosine-squared taper is necessary for the perturbed model to approach PREM smoothly at depth without introducing structural discontinuities in the outer core. The value of −0.5% is chosen after a series forward modeling tests. One of the test results is displayed in Fig. S4 in Supplementary Information. The model is on average −0.23% slower than PREM in the top 400 km of the outer core. In comparison with Fig. 2d, which shows that PREM is too fast in the topmost outer core, we conclude that a value of −0.5% is an appropriate bound for the model prior.

Bayesian inversion depends on making model inferences based on sufficient knowledge of *σ*(**m** | **d**), which requires that the model space be sufficiently sampled. A very common and straightforward approach to sampling the model space is the Markov Chain Monte Carlo (MCMC) sampler, which has been exhaustively documented in the literature. Here we use the MCMC enhanced by the Metropolis-Hastings algorithm^30^,^31^,^32^, which provides a mechanism of rejection and acceptance of the model samples to improve the efficiency in making the Bayesian inference.

Although the MH-MCMC algorithm provides an efficient scheme to conduct the Bayesian inversion, the number of model samples is still large. Since the predicted data **g**(**m**) is used to evaluate the likelihood function, for each model sample we need to run the DSM to calculate the synthetics for all earthquakes and measure the S3KS-SKKS differential travel times by cross-correlation, which would be impractical for the inversion where tens of thousands of models are sampled. Therefore, we use the much more efficient TauP Toolkit^33^ to predict the differential travel times. However, the ray-theoretical travel times calculated by the TauP Toolkit are slightly different from those measured by waveform cross-correlation due to the finite-frequency effect and possible interference of SKKS and S3KS waves with their neighboring phases, which must be corrected. This difference is mostly attributable to the fact that the SKKS and S3KS waves are not impulses, but have waveforms of limited width in time. Therefore, this finite-frequency effect does not depend on the particular model. So that we can estimate this difference by comparing the S3KS-SKKS differential travel times in PREM obtained by the TauP toolkit and those from cross-correlation of the DSM synthetics, and use it to make corrections to the TauP Toolkit results for the model samples in the inversion. The correction values estimated for PREM are plotted in Fig. S5 in Supplementary Information.

## Author Contributions

V.T. conducted most of the hands-on works including data processing, differential travel time measurement, forward modeling, and plotting. L.Z. was responsible for the overall design and operation of this research and wrote the manuscript. V.T. and L.Z. carried out the Bayesian inversion. S.-H.H. participated in the design of the research and was involved in the interpretation of the result and editing of the manuscript.

## Supplementary Material

Supplementary Information Supplementary Information
