# Supplementary material for: Seismological evidence for a non-monotonic velocity gradient in the topmost outer core
Source: Sci Rep. 2015 Feb 27;5:8613. doi: 10.1038/srep08613 (PMC4342554; doi:10.1038/srep08613)
Supplement: Supplementary Information [file srep08613-s1.pdf]

Supplementary Information for

Seismological evidence for a non-monotonic velocity gradient in the topmost outer core

Vivian Tang[1], Li Zhao[1] and Shu-Huei Hung[2]

1. Institute of Earth Sciences, Academia Sinica, Taipei, Taiwan
2. Department of Geosciences, National Taiwan University, Taipei, Taiwan

This Supplementary Information contains five figures.

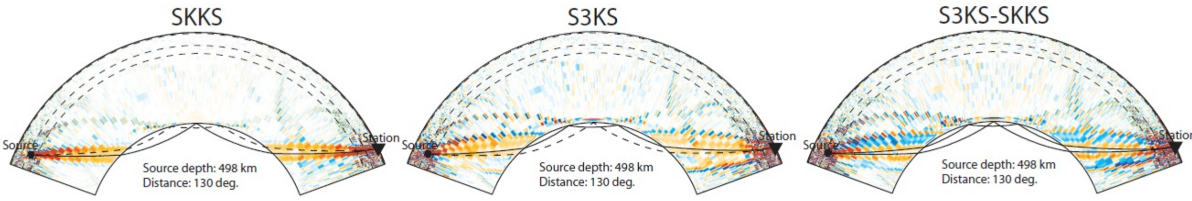

Fig. S1 | Sensitivity kernels. Shown here are sensitivity kernels of the travel times of SKKS and S3KS waves and their differential travel times to shear-wave speed at an epicentral distance of 130º for a source depth of 498 km. The kernels are calculated in model PREM by normal-mode summation[23] with an effective frequency band of up to 0.12 Hz (period longer than ~ 8 sec). Ray paths of SKKS and S3KS in PREM are also shown. At this distance, S4KS and S5KS arrive about 6 sec and 8 sec after S3KS, respectively, and have small contributions to the kernel of S3KS. Both SKKS and S3KS have broad first Fresnel zones and therefore the sensitivity of their differential time has a complicated pattern in the mantle (including in the D" region). Since our dataset has hundreds of S3KS-SKKS differential travel times with a diverse geographical coverage, it would require an extremely laterally complicated and physically implausible model to yield the dominate trend of positive residuals in our

S3KS-SKKS differential travel times. However, the complicated pattern of the sensitivity kernels for the differential travel times would certainly contribute to the scattering of our observations around this dominate trend.

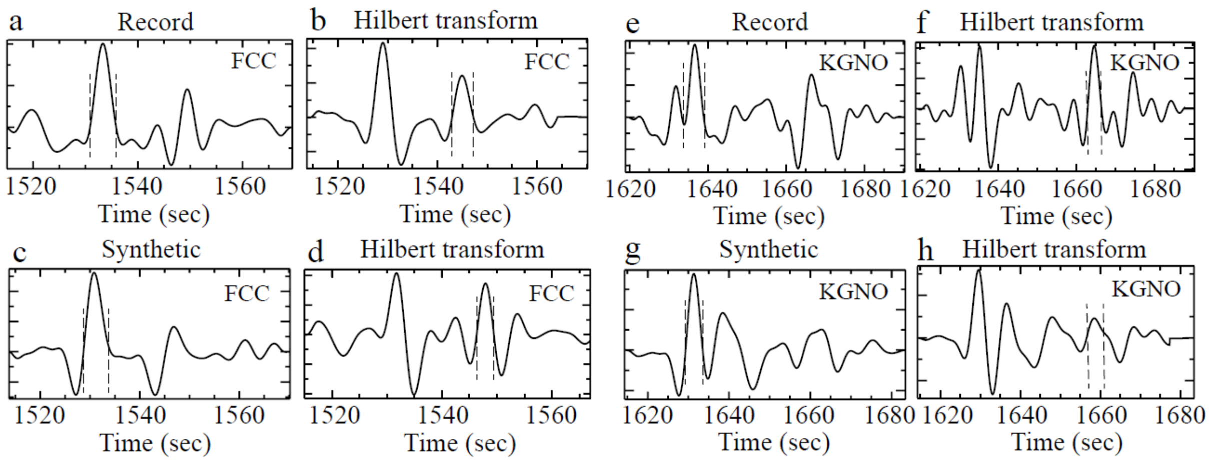

Fig. S2 | Quality control of S3KS-SKKS measurements. Two examples of differential travel time measurements of different quality at stations FCC (**a-d**) and KNGO (**e-h**) from the 25 July 2004 Sumatra earthquake. Shown are record at FCC (**a**), its Hilbert transform (**b**), DSM synthetic (**c**), and its Hilbert transform (**d**). The portions of the waveforms marked by the vertical dashed lines are examined and the similarity of the Hilbert-transformed S3KS waveform is an indication of the measurement quality. Waveforms at KNGO are plotted in (**e-h**) in the same way as (**a-d**). Both records and synthetics are used in quality control. The resemblance of a synthetic to its corresponding record is an indication of the effect of lateral heterogeneity along the path. One of the objectives of the quality control is to minimize the effect of lateral heterogeneity.

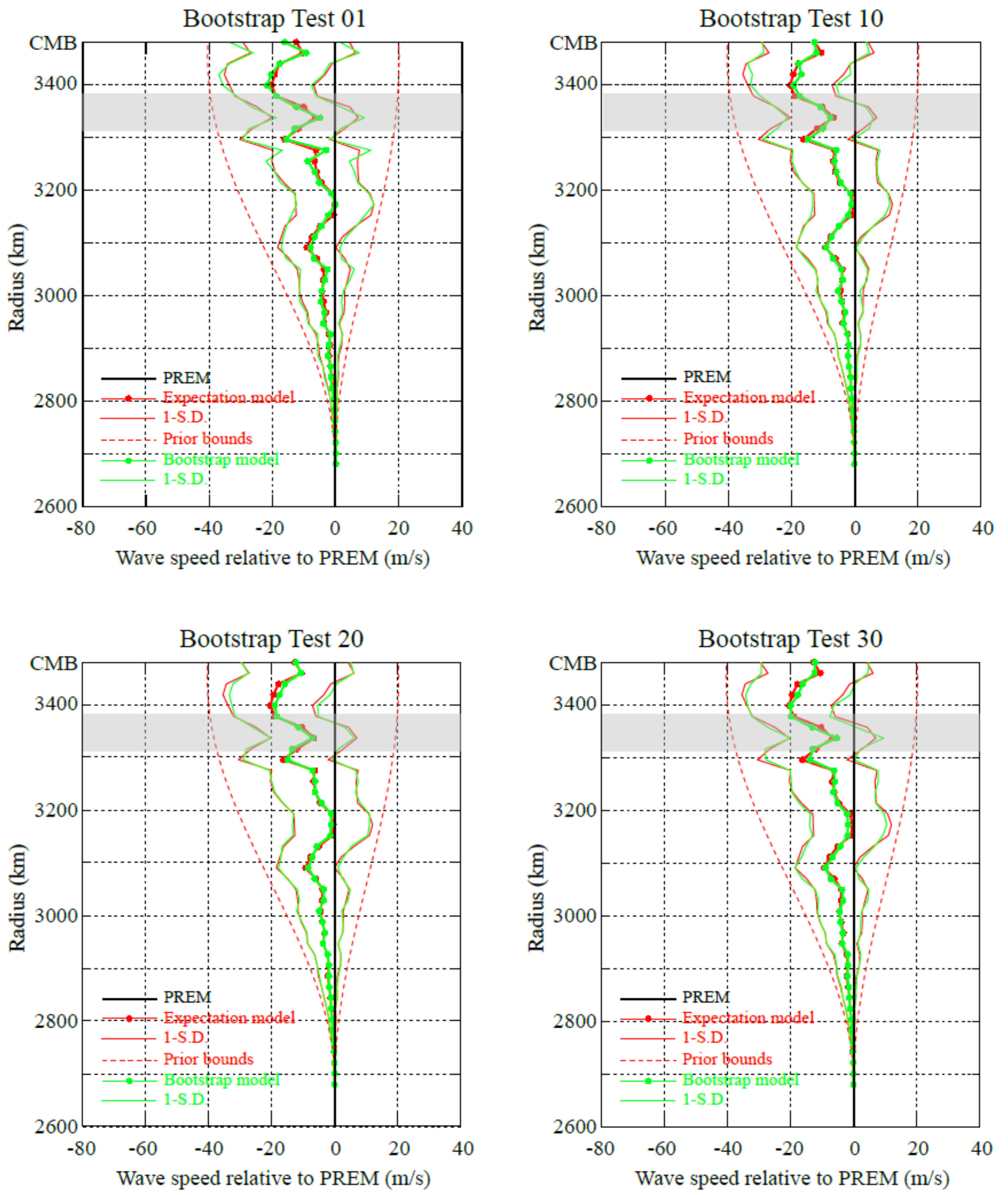

Fig. S3 | Bootstrap tests. Shown here are selected results from a series of bootstrap test inversions. In each inversion, 80% of the data are randomly selected and everything else remains the same as the inversion using all data. Red lines are the same as in Fig. 3(b). Green lines are for solutions from the bootstrap inversions.

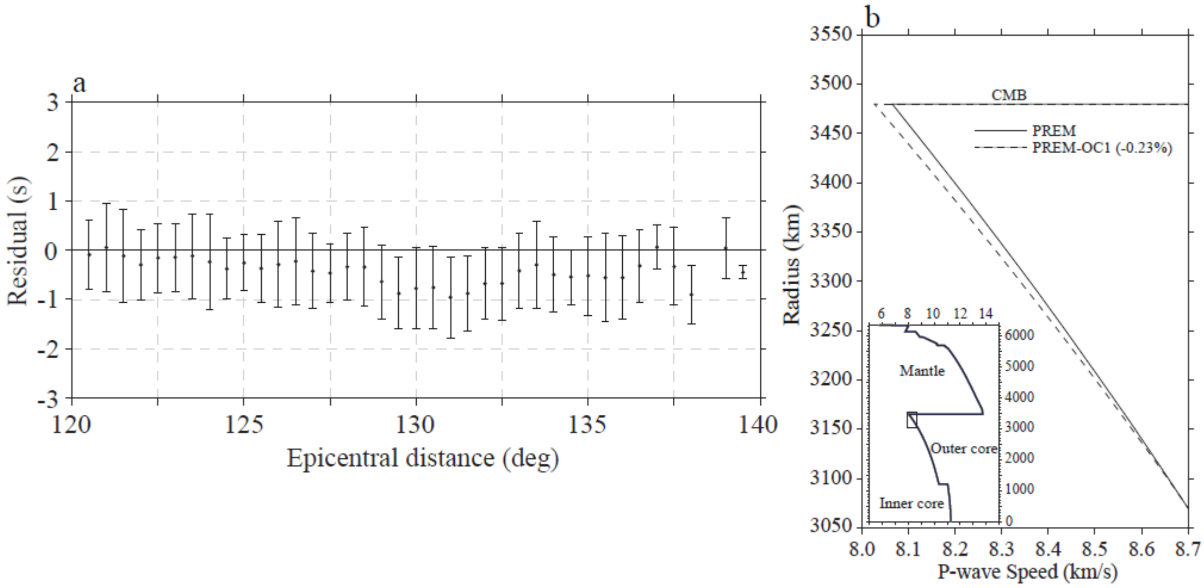

Fig. S4 | Modeling experiment of S3KS-SKKS differential travel times. In (**a**) we plot the residuals of the observed S3KS-SKKS differential travel times (Fig. 2b) relative to those predicted by a test model (PREM-OC1) whose structure is the same as PREM except for the top 400 km of the outer core where the wave speed is lowered by -0.23% on average. The fact that almost all of the residuals are less than zero indicates that the reduction in PREM-OC1 of -0.23% in the wave speed in the top outer core is too large. Therefore, we choose a lower bound of -0.5% perturbation from PREM for the model prior in our Bayesian inversion. The test model PREM-OC1 is compare with PREM in (**b**).

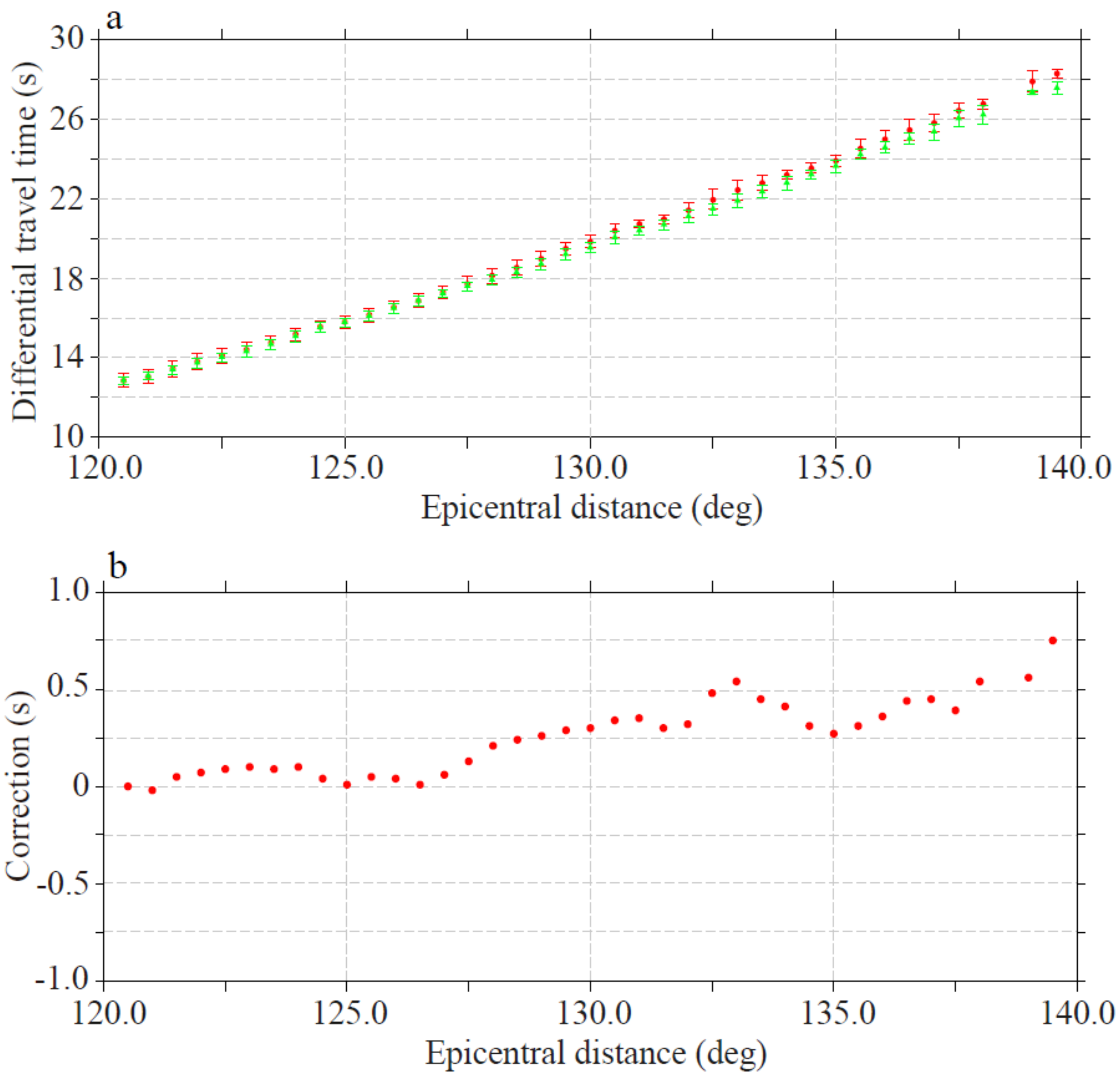

Fig. S5 | Corrections to differential travel times predicted by TauP Toolkit. We obtain the S3KS-SKKS differential travel times for PREM using both cross-correlations of DSM synthetics and the TauP Toolkit. They are plotted in (**a**) with red for cross-correlation results and green for TauP Toolkit ones. The differences between the two types of model predictions in (**b**) are used to correct for the finite-frequency effect in the predictions by the TauP Toolkit for other sampled models in the Bayesian inversion.
